# Experimental and Simulation Research on the Preparation of Carbon Nano-Materials by Chemical Vapor Deposition

**DOI:** 10.3390/ma14237356

**Published:** 2021-11-30

**Authors:** Bo Yang, Lanxing Gao, Miaoxuan Xue, Haihe Wang, Yanqing Hou, Yingchun Luo, Han Xiao, Hailiang Hu, Can Cui, Huanjiang Wang, Jianhui Zhang, Yu-Feng Li, Gang Xie, Xin Tong, Yadian Xie

**Affiliations:** 1 Faculty of Metallurgy and Energy Engineering, Kunming University of Science and Technology, Kunming 650093, China; gznuyangbo@163.com (B.Y.); hhouyanqing@163.com (Y.H.); 2 School of Materials and Architectural Engineering, Guizhou Normal University, Guiyang 550014, China; 3 Key Laboratory of Low-Dimensional Materials and Big Data, School of Chemical Engineering, Guizhou Minzu University, Guiyang 550025, China; glxprettylife@163.com (L.G.); xuexm0418@163.com (M.X.); wanghaihe1985@163.com (H.W.); lychun36@sina.com (Y.L.); hanx@gzmu.edu.cn (H.X.); hlhu@gzmu.edu.cn (H.H.); ccals@163.com (C.C.); whj2017@gzmu.edu.cn (H.W.); gszhangjh@126.com (J.Z.); 4 Guizhou Ecological and Environment Monitoring Center, Guiyang 550014, China; 5 CAS Key Laboratory for Biomedical Effects of Nanomaterials and Nanosafety, Institute of High Energy Physics, Chinese Academy of Sciences, Beijing 100049, China; 6 State Key Laboratory of Common Associated Non-Ferrous Metal Resources Pressure Hydrometallurgy Technology, Kunming 650503, China; 7 School of Chemistry and Materials Science, Guizhou Normal University, Guiyang 550014, China

**Keywords:** carbon nanotubes, carbon nano-materials, chemical vapor deposition, numerical simulations, reactor structure, chemical reactions

## Abstract

Carbon nano-materials have been widely used in many fields due to their electron transport, mechanics, and gas adsorption properties. This paper introduces the structure and properties of carbon nano-materials the preparation of carbon nano-materials by chemical vapor deposition method (CVD)—which is one of the most common preparation methods—and reaction simulation. A major factor affecting the material structure is its preparation link. Different preparation methods or different conditions will have a great impact on the structure and properties of the material (mechanical properties, electrical properties, magnetism, etc.). The main influencing factors (precursor, substrate, and catalyst) of carbon nano-materials prepared by CVD are summarized. Through simulation, the reaction can be optimized and the growth mode of substances can be controlled. Currently, numerical simulations of the CVD process can be utilized in two ways: changing the CVD reactor structure and observing CVD chemical reactions. Therefore, the development and research status of computational fluid dynamics (CFD) for CVD are summarized, as is the potential of combining experimental studies and numerical simulations to achieve and optimize controllable carbon nano-materials growth.

## 1. Introduction

Carbon is one of the most abundant elements in nature and is the basis of organic chemistry. Carbon materials can be easily produced by straightforward conversion reactions [1], and are commonly used in many fields due to their excellent electrochemical and thermodynamic properties. Over the past few decades, the discovery of carbon materials, for instance, carbon fiber, graphene, carbon nanotubes, and other carbon nano-materials, have provided unlimited possibilities for the development and utilization of carbon.

### 1.1. Carbon Fibers

Generally, fibers with a carbon content of more than 90% are called carbon fibers [2,3]. Carbon fiber has good mechanical properties, thermal conductivity, and electrical conductivity, as well as high stability and low density. In 1879, Edison first made carbon fiber from cellulose fibers, such as bamboo, flax, or cotton yarn. However, the properties of the fibers made at that time were not good, and the process could not be industrialized. Until the early 1950s, with the development of aerospace technology, there was an increasing demand for new materials with excellent properties such as carbon fiber, and the industrial production of carbon fiber was also developed [4].

**Figure 1:**
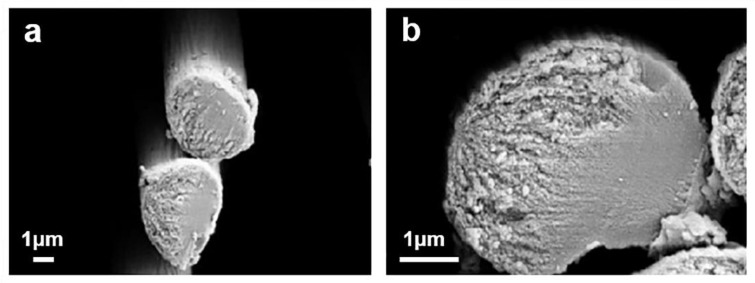
SEM images of carbon fibers, the magnification are (**a**) 10KX and (**b**) 30KX [7]. Copyright © 2021 Elsevier Ltd. and Carbon.

In the early 1960s, it was discovered that carbon fibers produced from polyacrylonitrile (PAN) had higher tensile and compressive strength [5]. At present, most carbon fiber is made of polyacrylonitrile. Cross-sectional images of PAN-based carbon fibers [6], are shown in Figure 1 [7]. Because carbon fiber has high strength but low density, it is often used in large devices such as airplanes and ships.

### 1.2. Graphene

Since Novoselov et al. separated off a single-layer graphene for the first time in 2004, its excellent performance has attracted the interest of researchers in various disciplines [8]. Single-layer graphene has the characteristics of high optical transparency, high electron mobility, high thermal conductivity, high mechanical strength, etc. [8,9,10,11]. Recently, researchers have discovered that better performance can be obtained by twisting graphene [12]. Graphene is considered as one of the most promising new materials because of its good properties and wide application [13]. 

**Figure 2:**
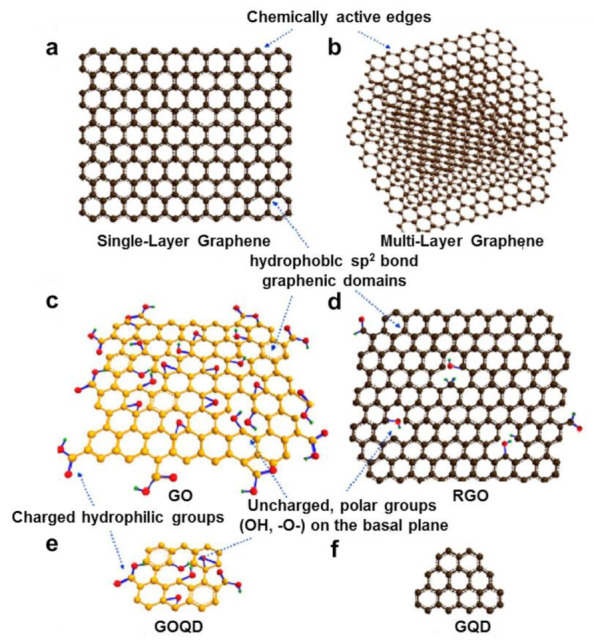
(**a**) Single-layer graphene, (**b**) multilayer graphene, (**c**) GO, (**d**) RGO, (**e**) GOQD, (**f**) GQD [19]. Copyright © 2021, The American Chemical Society.

Graphene is a flat two-dimensional structure formed by stacking up layers of carbon nanostructured graphite [14], as shown in Figure 2a. According to the stacking forms or the functional groups connected, graphene is divided into several different types, as show in Figure 2b–f [15,16,17,18]. 

Because of the excellent properties of graphene and its wide range of applications, it is urgent to prepare high-quality graphene with controllable structure, thickness, and size on a large scale. So far, there are many methods for preparing graphene, among which the commonly used methods are mainly mechanical exfoliation, chemical, and chemical vapor deposition (CVD). There will be an introduction later about preparing graphene by CVD.

### 1.3. Graphene-like Materials

**Figure 3:**
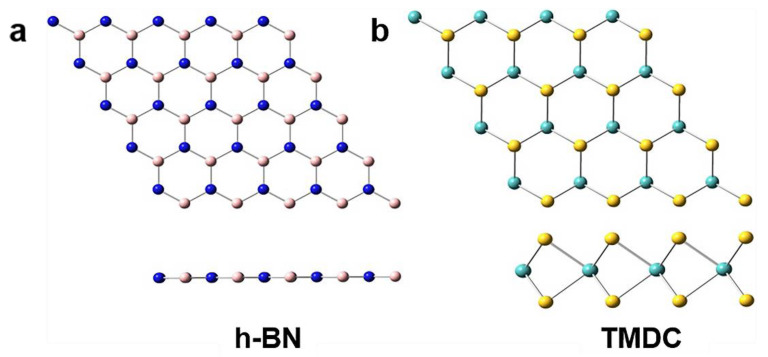
(**a**) Structure of h-BN layers and (**b**) TDMC [31]. Copyright © 2021 Elsevier B.V., Computational and Theoretical Chemistry.

As described above, graphene is a two-dimensional honeycomb lattice composed of single-layer carbon atoms. It is the basic component of a series of well-known carbon materials. The appearance of graphene has given researchers a lot of inspiration. They believe that two-dimensional layered materials have special and excellent properties. Therefore, there has been much research and discussion on a large number of emerging two-dimensional materials such as hexagonal boron nitride (h-BN), metal sulfides, and transition metal sulfides [20]. There are more than 40 types of transition metal dichalcogenides (TMDC), as shown in Figure 3. Like layered transition oxides [21,22], h-BN [23], and two-dimensional material topological insulators [24,25], they are all layered materials with a stacked structure.

The h-BN structure has a field-effect mobility comparable to that of graphene [26]. The MoS_2_ chip is a direct band gap semiconductor. These two structures, h-BN and MoS_2_, are potential graphene-like materials, which have great development prospects in many fields [27,28,29,30].

As a close relative of graphene, graphene-like materials have many properties similar to graphene, such as two-dimensional geometric properties with atomic thickness, good flexibility, excellent chemical stability, high thermal conductivity, and mechanical strength. Different from graphene, graphene-like materials have the characteristics of high temperature resistance, oxidation resistance, insulation, colorlessness, and so on. When the thickness of two-dimensional layered crystal is reduced to one or several layers, the electrical and optical properties of graphene-like structures may change significantly, and show properties that graphene does not have; because of this, graphene-like two-dimensional materials have become a research hot spot.

### 1.4. Carbon Nanotubes

**Figure 4:**
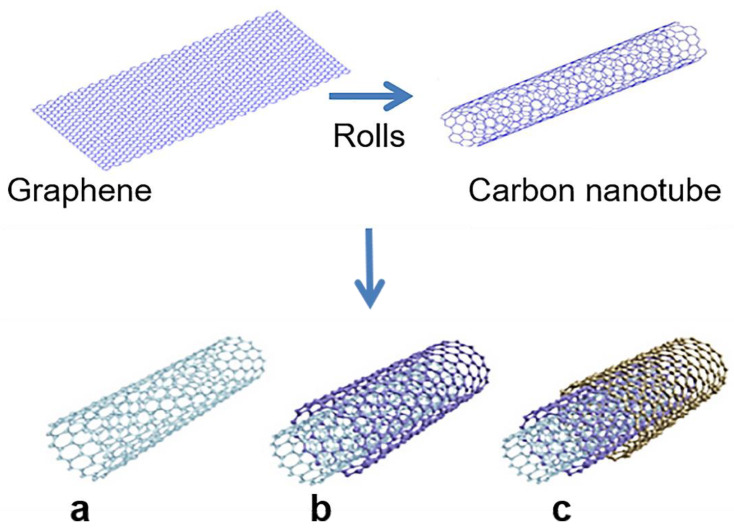
Diagram depicting carbon nanotube structures (CNTs) [38,39]: (**a**) single-walled CNTs; (**b**) double-walled CNTs; (**c**) multi-walled CNTs.

Carbon nanotubes are widely used due to their high aspect ratios, Young’s modulus, and electrical conductivity [32,33]. Geometrically, carbon nanotubes consist of a hollow tubular structure formed by several layers of graphite sheets wound along an axis. First discovered in 1952, carbon nanotubes (CNTs) were not identified as single-walled carbon nanotubes until 1976 [34]. CNTs were later described as single-walled carbon nanotubes (SWCNTs) in 1993 [35], and multi-walled carbon nanotubes (MWCNTs) were first discovered in 1991 by Iijima, as shown in Figure 4 [36,37,38,39].

**Figure 5:**
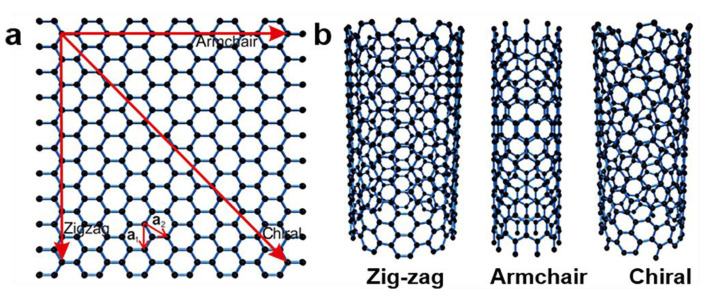
Different forms of single-walled carbon nanotubes (SWCNTs) [35]. (**a**) The chiral vector C determines the tube diameter; (**b**) the three structural forms of SWCNT structures.

Graphite sheets also have different curling angles, resulting in single-walled carbon nanotubes that can be categorized as zigzag, armchair, and chiral [40], as shown in Figure 5. New discoveries of different carbon nanostructure forms have prompted research for diverse applications. These materials have considerable application prospects in the fields of medicine [41,42,43,44,45,46], energy storage and conversion [47], sensors [48], and semiconductors [49]. Some applications have already begun to be realized. However, the specific carbon nanotube structure depends primarily on the preparation method.

Typical carbon nanotube preparation methodologies include arc discharging, laser ablation, chemical vapor deposition (CVD), and flame methods. Among these methodologies, CVD is a controllable and scalable method that can produce high-quality, large-area, two-dimensional materials at a reasonable cost. The basic principle of CVD is the decomposition reaction that occurs when the carbon-containing gas flows through the surface of a catalyst, generating carbon nanotubes on the surface of the catalyst [50]. In terms of product cleanliness and industrial scale, CVD is the most suitable method for producing carbon nanotubes. This method can also control the number of layers of our target product through certain means to maximize the practical value of the material.

The CVD process has the benefits of low cost, simple operation, and high product purity. However, many factors influence the CVD process, and the morphology of the final product is not easy to control. Because CNTs with different morphologies have different properties and applications, an in-depth study of nanostructured CNTs growth control factors is needed. Therefore, some important parameters that affect the quality of carbon nanotubes during the preparation process are also discussed later in this article. Examples include the carbon source and catalyst used in the preparation process and the control of some parameters (temperature, time, air flow rate, etc.) in the process [51].

Several studies have noted that the variable and difficult-to-control experimental conditions primarily affect the surface factors of CNTs, and local atmospheric changes in the reactor are the key to controlling the morphology of CNTs. Recently, in order to control the preparation of CNT materials, some researchers have started using computational fluid dynamics (CFD) software to simulate the gas concentration, temperature, and pressure distribution in the reactor, as well as the morphology of the nano- and micro-structured CNTs. 

This paper introduces how the morphology of the structure affects the properties of the materials and discusses in detail the preparation and simulation of carbon fiber, graphene, carbon nanotubes, and carbon nano-materials. The influence of chemical vapor deposition on the preparation of carbon nanotubes is primarily discussed. Additionally, the factors affecting the preparation of carbon nanotubes by chemical vapor deposition and the key results of carbon nanotube CFD simulation are discussed in detail.

## 2. Structure and Properties

### 2.1. Carbon Fibers

Commercial carbon fibers are typically 5–10 μm in diameter. Compared with glass fiber, because of its higher production cost, it does not get a large market. It was not until the end of the 20th century that carbon fiber began to be used in a variety of applications, with a downward trend in its price [5]. In recent years, more than 90% of carbon fibers are PAN fibers, which play an important role in making commercial carbon fibers, and the rest of carbon fibers are made from pitch.

In 1993, Kumar et al. found through experiments that the axial compressive strength of carbon fiber varies with the tensile modulus of the fiber and the precursor material required for preparation [52]. By comparing the structure and morphology of carbon fibers made from asphalt and polyacrylonitrile, they found that the width of the graphite sheet, the size of the microcrystals perpendicular to the fibers, and the anisotropy of the crystals play a significant role in explaining the difference in the compressive strength of carbon fibers. Huang et al. proved that carbon fibers made from pitch and polyacrylonitrile do have different microstructures [53]. Through research, it was found that the fiber structure made from pitch is relatively more uniform, whereas the fiber made from polyacrylonitrile has obvious differences but also has better arranged crystallites. In this way, people have a deeper understanding of the relationship between the structure of carbon fiber and mechanical properties.

The microstructure of carbon fiber is closely related to the precursor and processing conditions. Different models describing microstructure have been proposed. Perret et al. reported that voids have a preferred orientation in the fiber axis [4]. Watt and Johnson reported a branched microfiber structure in which most of the fibers are arranged along the fiber axis, and the width of these fibers is approximately 10 nm. Fourdeux et al. proposed a similar rayon carbon fiber fold band model. The average width of the banded monatomic layer is 6 nm and the length is approximately hundreds of nanometers. Needle-like micropores with a diameter of approximately 1–2 nm are enclosed in the plane of the stacked layer. The cross-section of traditional carbon fiber is circular. Modifying its circular surface profile to a more complex geometry requires controlling carbon etching or photolithography pattern carbon growth along the length of the fiber. Hunt et al. combined the spinning of bicomponent fibers and the carbonization of the required components with comparable properties to produce unidirectional, patterned, and continuous carbon fibers [54]. 

### 2.2. Graphene

**Figure 6:**
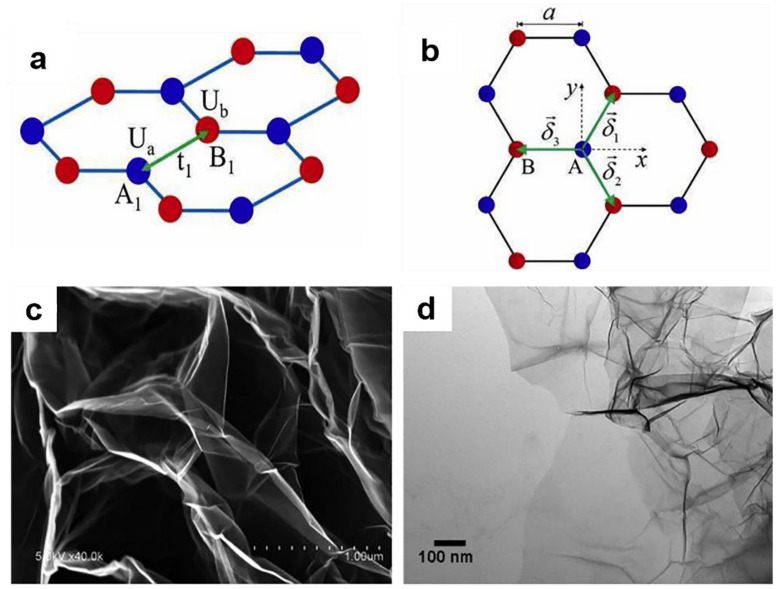
(**a**) and (**b**) Single-layer graphene lattice [56]. Copyright © 2021 Elsevier Ltd. and Solid State Communications. (**c**) SEM images and (**d**) TEM images of graphene [57]. Copyright © 2021 Published by Elsevier B.V. and Applied Surface Science.

Graphene has a two-dimensional cellular lattice structure composed of a layer of carbon atoms, which looks like the plane of a hexagonal grid [55] (Figure 6a,b). Each carbon atom hybridized with adjacent carbon atoms to form a regular hexagon, and each hexagonal unit is similar to a benzene ring. Each carbon atom contributes a bond free electron, and the graphene monolayer is only 0.335 nm. When being bent and deformed by an external force, the carbon atoms do not need to be rearranged to adapt to the external force, thus ensuring structural stability. The SEM and TEM images of graphene are shown in Figure 6c,d.

Due to the unique Dirac cone band structure of graphene near the Fermi level, it has high carrier concentration and ballistic transport. Graphene (known as zero-gap semiconductor) can also be wrapped in other graphite materials to expand its application range [58]. Many properties of graphene have yet to be explored. The nonlinear optical properties of graphene in solution and the hydrogen absorption properties of monolayer and bilayer graphene are important issues. In addition to experimental research, there is still considerable space for theoretical research on various aspects of graphene, including the dependence of electronic structure, doping effect, and properties on the number of layers [59].

### 2.3. Graphene-like Materials

Graphene-like materials are solids formed by stacking monatomic layers or polyhedral layers. The atoms or polyhedrons in the layers are connected with each other by covalent bonds or ionic bonds, and the layers are connected by van der Waals force. Due to the weak interlayer van der Waals force, the layered van der Waals solid can be stripped into several layers or even single layers, becoming a two-dimensional layered graphite-like material.

The structural lattice of BN and graphene carbon are similar, like a honeycomb structure composed of sp^2^ hybrids. (see Figure 3a) [60]. The coordination of transition metal atoms and chalcogen elements in the sandwich lattice of layered TMD crystals plays an important role. [20]. From the coordination and oxidation state of the metal atoms, it can be judged whether the layered TMD is a semiconductor or a metal [61]. Compared with graphite and h-BN, although the MoS_2_ layer is also composed of hexagons, its atomic connections are alternately connected by Mo and S_2_ atoms.

**Figure 7:**
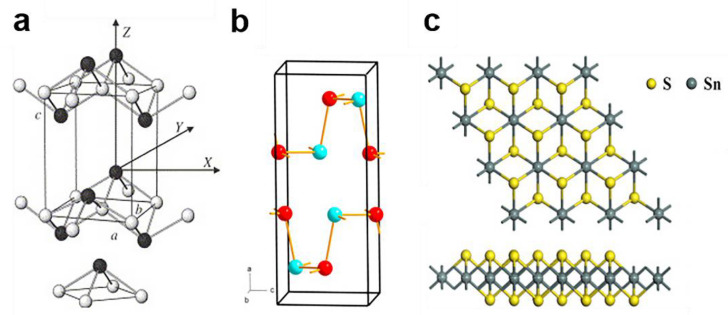
(**a**) Ball-and-stick model for litharge structure of SnO [64]. Copyright © 2021 Elsevier B.V. and Physica B: Condensed Matter. (**b**) The Pnma orthorhombic structure of GeSe [65]. Copyright © 2021 Elsevier B.V. and The Chinese Ceramic Society. (**c**) Top view and side view of SnS_2_ [66]. Copyright © 2021 Elsevier B.V. and Materials Chemistry and Physics.

In layered SnO (see Figure 7a), the structure is layered in the [001] crystallographic orientation with a van der Waals gap of 2.52 Å between the Sn1/2 −O− Sn1/2 sequence and adjacent Sn planes; in SnTe and PbX (X = S, Se, or Te), the formation of crystallization is a cubic NaCl structure; whereas GeTe and SnTe have a rhombohedral structure [62] (Figure 7b). The structure of SnS2 as shown in Figure 7c, in which Sn atoms are located in the octahedron between two hexagonal closely packed S plates to constitute a sandwich structure [63].

There are many kinds of graphene-like materials. Due to the layered structure, some graphene-like materials show better properties than graphene.

### 2.4. Carbon Nanotubes

**Figure 8:**
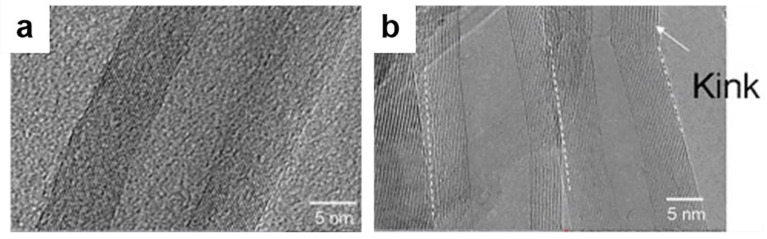
(**a**) HRTEM image of multi-shelled nanotube. (**b**) Multi-walled carbon nanotubes are welded together [68]. Copyright © 2021 Elsevier Ltd. and Carbon.

Carbon atoms can be combined in many ways to build structures with completely different properties. Several to dozens of concentric cylinders consisting of regular periodic interlayer spacing in MWCNTs are assembled at their centers. The interlayer spacing of MWCNTs is between 0.34 and 0.39 nm [67] (Figure 8). Both ends of MWCNTs are generally closed, and the ends are covered by dome-shaped semi-fullerene molecules (pentagonal defects), with axial dimensions ranging in size from 1 µm to 3–4 cm. The half-fullerene molecule (pentagonal ring defects) helps to close the cylinder at both ends.

Compared to MWCNTs, the diameter of SWCNTs is between 0.4 and 3 nm, and their lengths are usually in the µm range, and SWCNTs can be gathered together to form bundles (ropes). In the bundled structure, SWCNTs adopt a hexagonal organization, forming a crystal-like structure. Thus, SWCNTs are highly uniform and ideal molecular fibers. In addition, MWCNTs can be divided into Russian doll models and parchment models. The Russian doll model refers to the small CNTs nested in the largest CNTs, and the outer diameter is larger than the inner diameter. The model in which a simple graphene sheet rolls itself up like a roll of paper is called a parchment model.

Compared with SWCNTs, MWCNTs can be protected because they have a large number of carbon layers and have higher tensile strength.

CNTs have many excellent properties due to their unique structural characteristics. Studies have shown that the modulus of elasticity of carbon nanotubes can reach 1 Tpa [69,70], which is equivalent to that of diamond, and approximately five times higher than steel. The tensile strength of carbon nanotubes exceeds 5 GPa, exceeding the 2 GPa tensile strength of high-strength metals [71]. In addition, carbon nanotubes have good thermal and chemical stability and excellent thermal conductivity and superconductivity [72], and these properties, especially their optical properties [73], demonstrate that carbon nanotubes have significant research value and developmental potential.

Wang et al. [74] simulated and calculated the conductivity and percolation threshold of aligned carbon nanotube composites. The study found that the electrical conductivity and percolation threshold of the composite material was dependent on the length, aspect ratio, and structure of the carbon nanotubes, and more specifically, the geometric size and structure of the carbon nanotubes.

Hu et al. [75] studied the effects of ordered carbon nanotubes on the low-temperature crack resistance of composite materials at low temperatures. The carbon nanotubes were oriented in a magnetic field, where the carbon fiber composite was prepared using the paramagnetic particles of ferroferric oxide, and the carbon nanotubes were aligned. The low-temperature mechanical properties of the composite material improved, and the thermal expansion coefficient decreased. Compared with pure resin, the thermal expansion coefficient of the carbon nanotube-oriented composite material was reduced by 41.6%, and compared with a carbon fiber resin composite material, the microcrack density at low temperatures was reduced by 56.2%.

Li and Dong [76] prepared epoxy resin/carbon nanotube composites using an electric field induction method. The carbon nanotubes were uniformly dispersed and arranged in the epoxy resin in an orderly manner along the electric field direction. When the content of carbon nanotubes was 2.5%, the tensile strength of the material increased by 26.3%. For carbon nanotubes, their structures may have defects or doped structures in addition to the common single wall and multi wall forms. Table 1 lists the effects of defects or doping on the properties of carbon materials.

For all carbon materials, in addition to the conventional structure, there will be defects, doping, and other structural differences. For different carbon materials, the effects of defects and doping are different. For example, when carbon fiber has defects, its mechanical properties will decline accordingly, but for MOS_2_, the effect of defects on its mechanical properties is positive. We list the main performance changes of some defects or doped materials reported in recent years in Table 1. In addition to the properties noted in the table, defects and doping may also have a certain impact on the catalytic and photothermal properties of the material. Therefore, mastering the preparation methods and repair methods of carbon materials is an important breakthrough in its application field.

## 3. Preparation of Carbon Materials

### 3.1. Carbon Fibers

There are many methods to prepare carbon fibers, such as chemical vapor deposition (CVD), sol-gel, electrospinning, and so on. Compared with the electrospinning method, the CVD method is used less often for carbon fiber preparation. With the development of CVD, the properties of CVD-prepared carbon fibers or composite carbon fibers are getting better and better.

**Figure 9:**
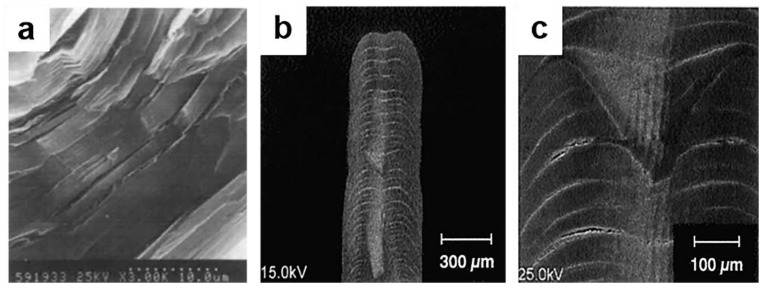
(**a**) SEM of the graphite fiber prepared by CVD of cyanoacetylene at 3000 °C on carbon fiber [107]. Copyright © 2021 Elsevier B.V. and Synthetic Metals. (**b**) Fiber profile, and (**c**) stripe microstructure at center of fiber [108]. Copyright © 2021 Elsevier Ltd. and Carbon.

In 1985, Matsumura et al. prepared highly conductive graphite fibers by cracking cyanoacetylene on the surface of carbon fibers and then performing heat treatment, and their conductivity remained stable for a period of time. The scanning electron microscope image is shown in Figure 9a [107]. In 1993, with methane as the precursor, the pressure in the reaction chamber greater than 1 bar, and the growth rate greater than 0.3 mm/s, a carbon fiber with good performance was obtained. [108]. That study provides some guidance for the preparation of carbon fiber by the CVD method.

V.K.Varadan et al. reported that the use of microwave CVD may avoid the use of toxic impurities contained in traditional methods [109]. Kang et al. observed a lamellar microstructure parallel to the deposition surface in carbon fibers prepared by methane laser CVD [108]. As shown in Figure 9b, when fibers were deposited at peak temperatures higher than approximately 2100 °C, the deposition center is usually concave, i.e., the tip of the fiber is similar to a volcano. From longitudinal section micrographs shown in Figure 9c, it can be observed that there is a striped structure in the central part of the fiber along the axial.

In recent years, some people have started to combine chemical vapor deposition with other fiber preparation methods (electrospinning, wet spinning, etc.) to prepare modified fibers. Feng et al. combined electrospinning technology with CVD to prepare superhydrophobic carbon fibers (SCFS), which still have superhydrophobic properties at 97 ℃ and maintain excellent superhydrophobic properties after being immersed in strong acid, strong alkali, and organic solvent for 120 days [110]. For long-term reuse under various harsh conditions, they have promising application.

**Figure 10:**
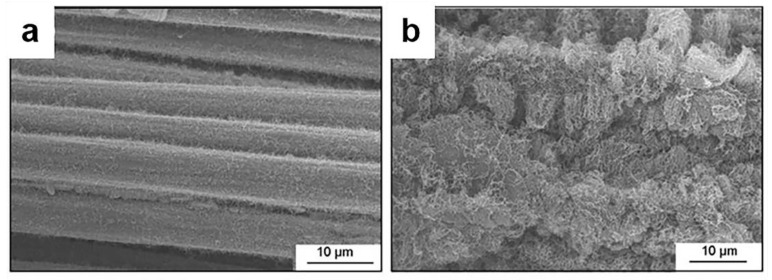
SEM images of the carbon fiber grown with (**a**) traditional thermal CVD and (**b**) modified thermal CVD [113]. Copyright © 2021 Elsevier B.V. and New Carbon Materials.

The CVD process can also be used for the modification of carbon fibers. Li et al. modified carbon fiber by CVD [111]. Rong et al. attached carbon nanotubes onto carbon fibers to increase the effective area. Figure 10 is an SEM image of a layer of CNTs coated on a carbon fiber by a CVD method [112].

### 3.2. Graphene

Currently, there are many different processes for preparing graphene, such as mechanical exfoliation, chemical methods [114], SiC graphitization epitaxial growth [115], and chemical vapor deposition (CVD) [116]. Usually, the CVD method uses copper as the substrate for growing grapheme [117]. CVD is one of the commonly used methods in the preparation of graphene; it can prepare high-quality graphene on a large area.

**Figure 11:**
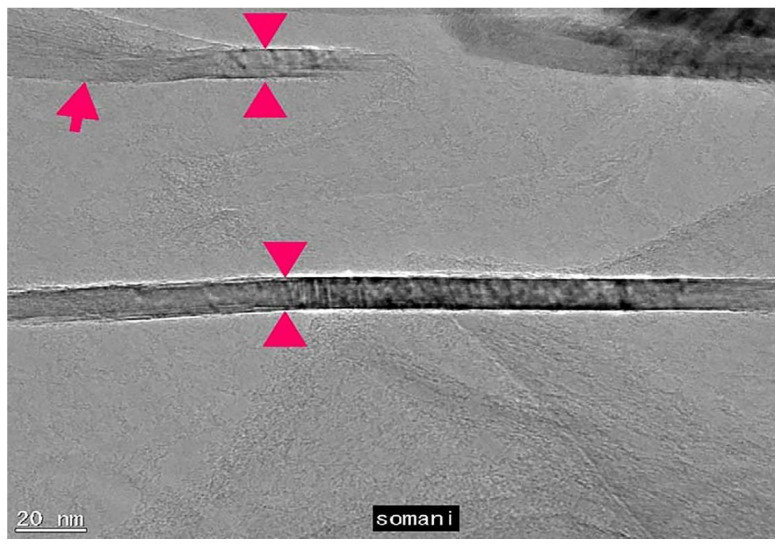
HR-TEM image of PFLG film [120]. Copyright © 2021 Elsevier B.V. and Chemical Physics Letters.

In 1975, the deposition of single-layer graphite material on Pt by CVD was first reported [118]. Subsequently, Eisenberg and Blackley reported the formation of a graphite layer on nickel (111) [119]. In 1984, researchers carried out IR, for what may be the first graphene grown on the metal surface [86]. In 2006, camphor (terpenoids, white transparent solids with the chemical formula of C_10_H_16_O) was first tried as precursor material. Figure 11 shows TEM image of planer few layer graphene (PFLG) film [120]. 

CVD has many different processes, which can be divided into hot wall, cold wall, or plasma enhancement (PECVD) according to the required materials, precursors, and structures. Graphene is mainly formed on copper film through a cold wall system.

Recently, Soriadi et al. reported the growth of graphene for the varied thicknesses of 100–600 nm by using a cold-wall CVD reactor [121]. Das et al. [122] used cold-wall chemical vapor deposition to prepare graphene, changing the nucleation density and average size by changing the growth parameters. They found that when the temperature was 1000 ℃ and the pressure was 700 Torr, reducing the ratio of CH_4_: H_2_ or reducing the concentration at a fixed ratio would reduce the growth rate of graphene films. The change of the CH_4_: H_2_ ratio had the greatest effect on the nucleation density. It was also found that the boundary morphology of graphene microcrystals depends on the growth conditions; with an increase in effective carbon deposition rate, the process shifts from irregular/serrated to convex hexagon to regular hexagon. This observation shows that the diffusion of C adsorbed atoms along the edge of the growing graphene microcrystals may play an important role in the morphological evolution of graphene nuclei.

Through experiments and computational fluid dynamics (CFD) simulation, Jia et al. found that the cold-wall CVD system can inhibit the gas phase reaction more than the hot-wall CVD system and can produce super clean graphene [123]. In contrast, the cold-wall system reduces the gas phase temperature because the heating is concentrated on the Cu substrate, which inhibits the formation of amorphous carbon. The reduction of amorphous carbon can be clearly seen by TEM.

In the future, the industry will emphasize the uniformity, size, growth rate, and transferability of graphene films, and its feasible mass production methods will be widely discussed in terms of capacity, output, cost, equipment, and industrial scale production line. The challenge of how to use simulation to obtain the optimal process conditions and commercialization of large-scale production still needs to be solved. Because the availability of clean surfaces will improve the performance of graphene for CVD and promote the discovery of new applications, the development of ultra-clean graphene will be more widely discussed in the future.

### 3.3. Graphene-like Materials

The preparation methods of graphene-like materials are the same as those of graphene. Mechanical stripping and liquid phase stripping are two of the commonly used methods in the preparation of layered samples. Separate sheets are separated from stacked two-dimensional layered crystals by breaking the weak van der Waals bond between layers. However, in order to synthesize two-dimensional layered materials on a large scale, CVD has been favored by researchers, which is expected to control the number of layers and crystal structure.

Müller et al. reported the growth of hexagonal boron nitride (h-BN) on a Pt surface as indicated by low-energy electron diffraction (LEED) [124]. In 2011, Sun et al. directly synthesized a carbon film with graphene properties through a chemical vapor deposition method at a temperature of 1000 °C without any metal catalyst [125]. The film thickness can be controlled by modifying the growth conditions.

Shi et al. [126] used atmospheric chemical vapor deposition to synthesize hexagonal boron nitride (h-BN) films and proved that the h-BN film followed the morphology of the nickel surface after growth. Qin et al. [127] chose to use CVD to synthesize h-BN films under ambient pressure. Most of the samples prepared by this method are composed of several atomic layers, but a small amount of single-layer h-BN flakes are also obtained. It can be concluded that the size of the h-BN sheet is approximately 0.8–2.5 μm. This method has great potential for mass production of a small amount of atomic layer boron nitride sheets. 

**Figure 12:**
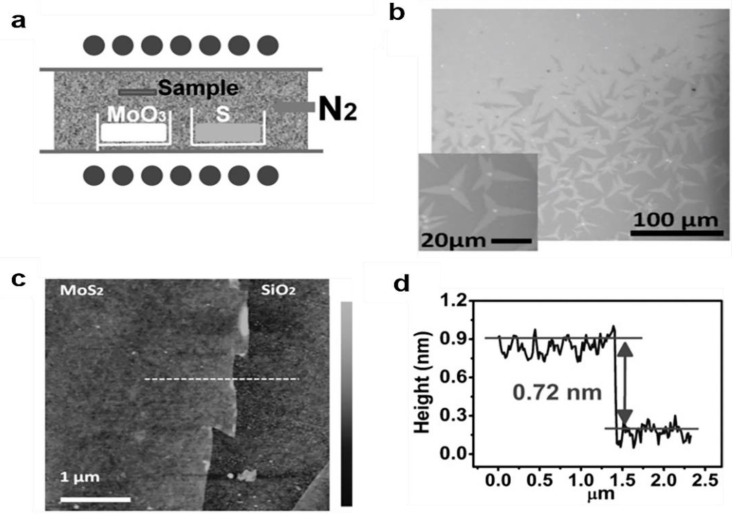
(**a**) Schematic illustration for the experimental set-up; (**b**) HRTEM image of MoS_2_ monolayer; (**c**) enlarged HR-TEM image of the marked area in figure (**c**) with an inset showing the SAED pattern; (**d**) in-plane XRD result for the MoS_2_ monolayer [128]. Copyright © 2021 WILEY-VCH Verlag GmbH & Co. KGaA, Weinheim.

Three-dimensional (3D) MX_2_ compounds display important properties such as semiconductivity, half-metallic magnetism, or charge density wave [61]. Lee et al. used MoO_3_ and S as reactants and successfully synthesized an MoS_2_ layer on SiO_2_ by chemical vapor deposition. The characterization also proved that the MoS_2_ synthesized by this method has high crystallinity (Figure 12b,c) [128]. The in-plane X-ray diffraction (XRD) profile for the MoS_2_ monolayer synthesized by the CVD method is shown in Figure 12d. This method can also grow other transition metal dihalides.

**Figure 13:**
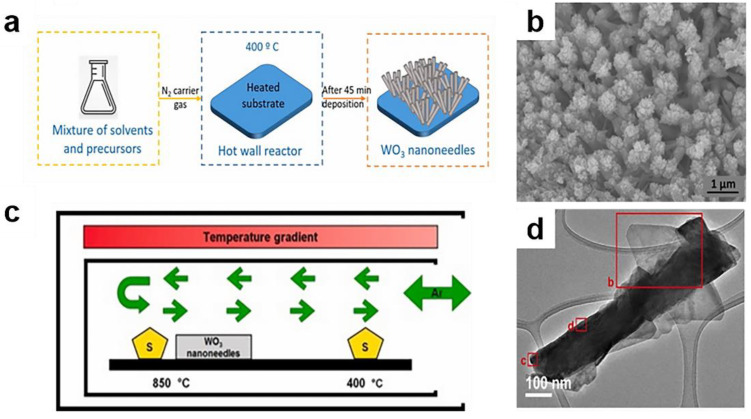
(**a**) AA-CVD synthesis of WO_3_ nanomaterials; (**b**) CVD synthesis of WS_2_ nanomaterials; (**c**) SEM images of WO_3_ nanorods; (**d**) TEM image of WS_2_ nanoneedles. [129] Copyright © 2021 Elsevier B.V. and Sensors and Actuators B: Chemical.

Alagh was the first to propose that aerosols can be used to assist the chemical vapor deposition method (AA-CVD), combining it with the CVD method without H_2_ atmospheric pressure, to realize the simple synthesis of two-dimensional layered WS_2_ nanosheets on the one-dimensional WS_2_ nanostructure [129]. The experimental steps are shown in Figure 13a and c: first, WO_3_ or nanoneedles are directly deposited on alumina substrate, and then vulcanization treatment is carried out. Figure 13b shows the SEM image of the material before vulcanization. Vulcanization promotes the growth of WS_2_ (Figure 13d).

So far, in addition to TMD, there are few studies on metal sulfides, and the technology for large-scale continuous production of two-dimensional materials has yet to be developed. Therefore, the most important task at present is to develop and realize large-scale production of graphene-like materials. From the existing methods, in addition to stripping, chemical vapor deposition and chemical methods are very promising. However, there are few studies on the preparation of graphene-like materials by combining simulation and chemical vapor deposition. If it can be used a little, it is very helpful to prepare graphene-like materials with good quality.

### 3.4. Carbon Nanotubes

When choosing a method to prepare the CNTs, CVD methods are particularly attractive because they can be easily scaled up when strict, custom control of the deposit structure is required. Commonly used carbon sources are CH_4_, carbon monoxide and acetylene. Carbon nanotubes of different structures can be obtained according to the adjustment of temperature and pressure. In this section, the factors affecting the preparation of carbon nanotubes by chemical vapor deposition are reviewed from three aspects: catalyst and support material, carbon precursor, and depositional conditions. 

#### 3.4.1. Catalyst and Support Material

**Figure 14:**
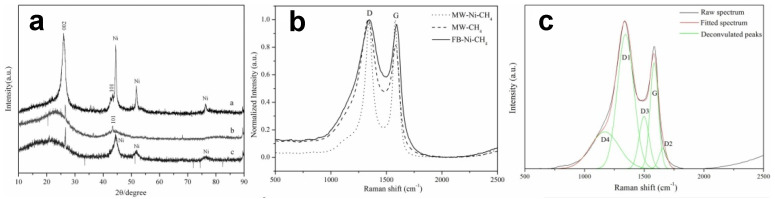
(**a**) X-ray diffraction pattern of pine nut shell (PNS) coke at 600°C: (i) nickel-containing catalyst during microwave radiation, (ii) nickel-free catalyst during microwave radiation, and (iii) nickel-containing catalyst in fixed bed; (**b**) normalized Raman spectra; (**c**) typical deconvoluted Raman spectra of carbon nanotubes (CNTs) [130]. Copyright © 2021 Elsevier B.V. and Diamond and Related Materials.

Zhang et al. [130] used CH_4_ as a carbon source and nickel as a catalyst to form tubular and multi-walled carbon nanotubes. In their research, they found that at 600 °C, the formation of carbon nanotubes is the greatest. Microwave radiation and nickel catalyst play key roles in the growth process. Through X-ray diffraction (XRD) and Raman analysis (Figure 14), the resulting carbon nanotube structure is perfect, and the growth process follows the cutting-edge growth mechanism.

**Figure 15:**
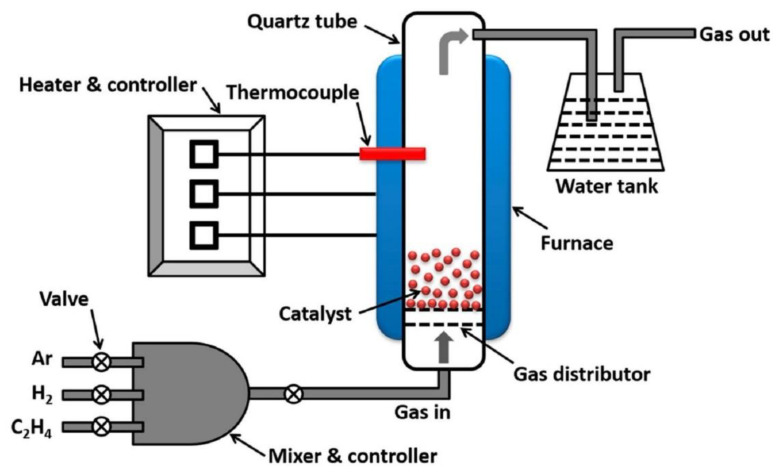
Schematic of fluidized bed CVD setup [131].

Shukrullah et al. [131] synthesized multi-walled carbon nanotubes by cracking ethylene gas molecules in a fluidized bed CVD reaction chamber with Fe_2_O_3_/Al_2_O_3_ as composite catalyst; the schematic diagram of the process is shown in Figure 15. They calculated the adsorption capacity of carbon nanotubes and amine functionalized carbon nanotubes from the penetration curve of carbon nanotubes in the growth state. In other words, among all the tested samples, the CO_2_ adsorption capacity of amine-functionalized MWCNTS is the highest, approximately 129 cm^3^/g.

Wang et al. [132] discovered that the addition of iron in the catalytic process will lead to the negative charge of the grown carbon nanotubes; in addition, they also found that if different external electric fields were applied, the chirality of carbon nanotubes would be significantly affected by the change of Fermi level of iron catalyst.

In 2016, Li and Zhang [133] used Fe-Cu catalysts to selectively grow single-walled and multi-walled carbon nanotubes. By introducing a certain amount of Cu to inhibit the diffusion of Fe nanoparticles, the growth of single-walled carbon nanotubes can be realized; multi-walled carbon nanotubes can then be obtained by introducing a Cu catalyst on Fe/Al_2_O_3_.

In 2018, Shah et al. [134] reported the growth of MWCNTs on a Pt–W catalyst at 800 °C using the CVD technique. In the CVD system, the growth of nanotubes is usually controlled by the size of catalyst particles. If the catalyst particle density is high and the catalyst activity is widely distributed, linear and wavy multi walled carbon nanotubes are formed, whereas the formation of spiral/curly multi walled carbon nanotubes is usually explained based on the interaction between specific catalyst particles and growing nanostructures. The HRTEM images clearly revealed MWCNT formation with no lateral carbon deposits. Raman analysis also suggested that the concentration of defective molecules was low in the sample. It provides a basis for the new research field of carbon nanotube synthesis and its application in the electronic industry.

Presently, researchers have proposed a variety of purification methods for carbon nanotubes, which can be roughly divided into two methods: physical and chemical. The physical method mainly separates impurities and carbon nanotubes based on their physical properties. For example, single-walled carbon nanotubes prepared by arc or laser methodology are purified by microfiltration, and the final product is separated into pure SWNTs, fullerenes, carbon nanospheres, and polyaromatic carbon [135]. Spatial exclusion chromatography (SEC) is also commonly used as a physical purification method [136], with the advantages of high efficiency and fast separation, and this method is expected to be widely implemented in actual production. The chemical method primarily uses carbon nanotubes and different reactions of impurities and oxidizing substances to achieve purification. Common methods include dry oxidation [137], wet reflux oxidation [138], high temperature hydrogen treatments [39,40], and graphite intercalation compounds [139]. Zhao et al. found that the content of nanotubes increased when monitored by scanning electron microscope in the purification process with an increase in purification [137]. Magnetism was studied by using electron spin resonance (ESR). Two samples are used in electron spin resonance measurements of MWNT and SWNT; one is dispersed in hexane to form loosely contacted tubules, and the other is dried deposited tubules to achieve a tightly contacted tubule state. By using these samples, it was found that the electron spin resonance alignment is closely related to the contact between nanotubes. However, the chemical purification method can cause defects in the carbon nanotube structures during treatment, limiting the production of terminal and intertwined products.

Many impurities, such as amorphous carbon and metal particles, may be introduced in the process of synthesizing carbon nanotubes. Therefore, the purification stage in the late preparation stage is still very important to the purity of the product.

**Figure 16:**
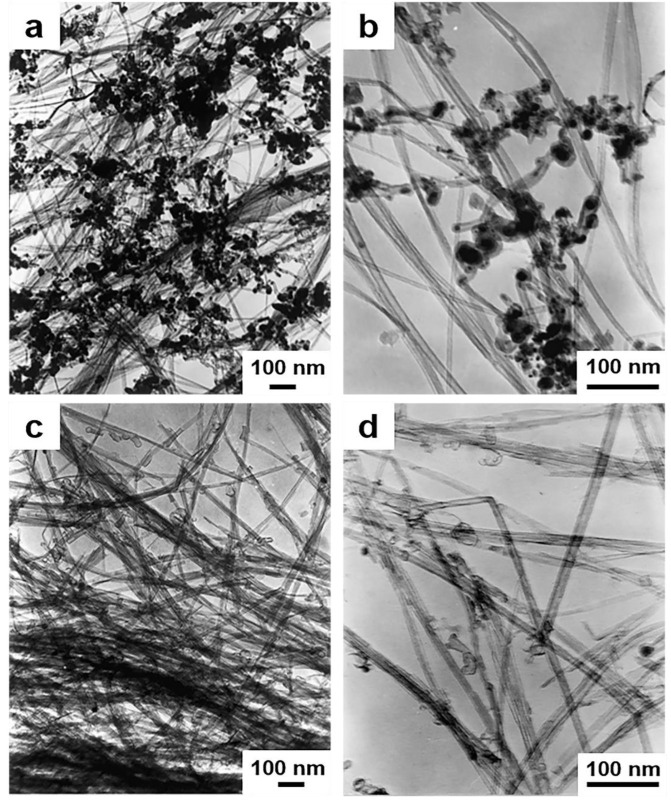
TEM images of the as-synthesized MWCNT product (**a**), the purified MWNTs without bromination (**b**), the purified MWNTs with bromination (**c**), and the purified MWNTs with bromination (**d**), showing some of the MWNT tips [145]. Copyright © 2021 Elsevier Science B.V. and Carbon.

There are many reports on the purification method of CNTs. One of the most effective methods is to maintain a temperature of 750 °C for oxidation in an ordinary atmospheric environment. For pure CNTs, the oxidation stage is crucial. The use of KMnO_4_/H_2_SO_4_ mixed solution for liquid phase oxidation is also a feasible purification method. Although this method can obtain ultra-pure carbon nanotubes, the carbon nanotubes may be severely damaged in the end. Some have also proposed a purification method for graphite intercalation. According to previous studies, the bromine resistance of carbon nanotubes is not as good as that of carbon nanoparticles [140,141,142,143,144]. Hou et al. [145] proposed a process for efficient purification of carbon nanotubes and improved the multi-step purification process. The first step of this purification process is to obtain well dispersed nanotube structures, and the raw materials were subjected to ultrasonic and heat treatment. They were then soaked in bromine water at 90 ℃ for 3 h and kept heated in the open air for 45 min to 520 ℃. Finally, the remaining powder was immersed in 5m hydrochloric acid at room temperature to remove metal particles. The TEM image of the final synthesized product is shown in Figure 16.

One of the biggest purification challenges is the purification of single-walled carbon nanotubes (SWNTs). Strong et al. studied a simple and relatively cheap SWNTs purification method [146]. Their method includes two steps: oxidative heat treatment and pickling. The purpose of the oxidation heat treatment stage is to oxidize and pickle the metal catalyst to achieve the purpose of removing the oxidant [147]. Li et al. [148] synthesized SWCNTs through the catalytic decomposition of hydrocarbons, and their analysis showed that the resulting product had amorphous carbon and catalyst particles. They then ensured that the vapor-grown carbon nanofibers and multi-walled carbon nanotubes are removed from SWCNTs without damaging them. The yield of SWCNTs purified by this method is 40%, and the purity is approximately 95%.

Chattopadhyay et al. [149] used a one-to-one mixture of hydrofluoric acid and nitric acid to purify the SWNT soot obtained by ultrasonic treatment. Dillon et al. [144] used hot-water to treat partial oxidation and HCl solvent oxide at 700 °C, but some amorphous carbon and metal particles were still observed. Therefore, the removal of the metal catalyst by heating the material to a temperature at which the metal can evaporate is also a purification method [147]. High-temperature annealing can also remove the surface oxygen of the carbon nanotube material to achieve purification [150]. Andrews et al. [151] proposed that high temperature annealing is an effective way to purify carbon nanotubes. Chen et al. [152] reported a three-step process to purify multi-walled carbon nanotubes using Ni-Mg-O as a catalyst. For the third purification step, 510°C was chosen as the optimal nanotube burning temperature for eliminating non-nanotube carbon materials. This temperature was consistent with the results obtained by Colomer et al. [136], who determined that 500 °C was the optimal temperature. The purity of the purified MWNTs is more than 96% without damage. Biró et al. [153] used dry and wet chemical methods to remove unwanted catalysts. However, wet oxidation will cause relatively moderate damage to the outer wall of the carbon nanotubes, and because the metal particles are wrapped in the center of the carbon nanotubes, they cannot be removed even if the treatment is repeated.

So far, the method of purifying carbon nanotubes is still being discussed. Choosing an appropriate purification method is very important for the later application of carbon nanotubes. At the same time, a purification method with simple operation, low price, low energy consumption, and high purification efficiency is being explored and pursued by everyone.

#### 3.4.2. Carbon Precursor

A traditional CVD technique was developed to produce a high CNT yield by multiplying the reactors in mostly three-quartz tubes [154]. In that design, the preparation of carbon nanotubes is achieved by single or multiple quartz tubes. Using high-purity CH_4_ as a carbon source and Fe as a catalyst, carbon nanotubes with an average diameter of 30 and 24 nm were produced at 650 °C. This design not only improves the yield and quality, but also reduces the loss of energy consumption.

Another investigation assessed the gaseous and condensate [155] products of ethanol decomposition during the synthesis of SWCNTs by aerosol chemical vapor deposition (floating catalyst CVD). Analysis shows that using CO and CH_4_ as a mixed carbon source can produce relatively pure carbon nanotubes. The yield of carbon nanotubes produced by catalytic CO disproportionation is higher. Research has found that carbon deposition on the reactor wall plays an important role in the process of preparing carbon nanotubes.

**Figure 17:**
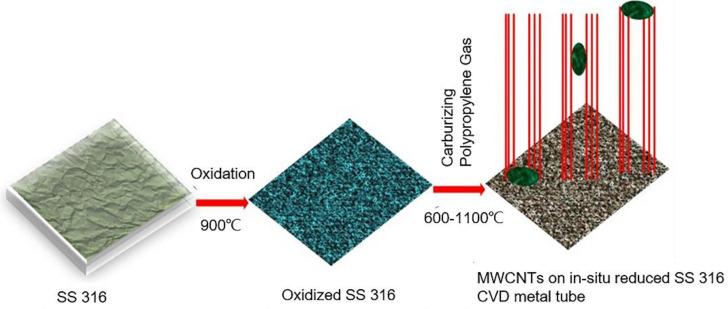
Mechanism of multi-walled carbon nanotube (MWCNT) growth using plastic waste and a stainless steel 316 (SS 316) metal tube [156].

Tripathi et al. [156] found that carbon nanomaterials can be made from plastic waste. As shown in Figure 17, plastic waste and SS316 metal tubes are used to grow carbon nanotubes, whereas the laboratory uses a centrifuge tube made of polypropylene as a carbon source. The metal tube can be used as a reactor or as a catalyst, and carbon nanotubes of good quality can be prepared under given conditions. 

Recent studies have shown that acetonitrile reversibly modulates the SWCNT diameter during CVD growth [157]. The researchers proposed non-equilibrium quantum chemical molecular dynamics simulations to explain the mechanism of this phenomenon. When single-walled carbon nanotubes nucleate, free radicals derived from acetonitrile will actively absorb hydrogen from the surface of the hydrocarbon. This process will reduce the overall surface carbon density; the release of hydrogen during the nucleation process accelerates the single-walled carbon. The nucleation kinetics of the nanotubes combined with the lower surface carbon density results in single-walled carbon nanotubes with a narrower diameter in the presence of acetonitrile.

Li et al. [158] proposed that the formation of carbon nanotubes depends on the chemical structure of hydrocarbon precursors. They believed that aromatic molecules are conducive to the preparation of single-walled carbon nanotubes, whereas aliphatic molecules form multi-walled carbon nanotubes. However, the formation of single-walled carbon nanotubes or multi-walled carbon nanotubes is not only dependent on the carbon source, but also closely related to other growth conditions (such as catalyst type, temperature, and gas flow rate).

#### 3.4.3. Depositional Condition

Polycrystalline carbon nanowires (P-CNWs) [32] have been synthesized using CVD with C_2_H_4_ as the carbon source and Ni particles distributed in porous Si_3_N_4_ ceramics as the growth seeds at 700 °C. The obtained P-CNW shows that the solid core and base surface of the graphite nanosheets are perpendicular to the spool. In addition, the elevated growth temperature promotes the formation of hollow carbon nanotubes at 750 °C and amorphous carbon above 800 °C, thereby changing the conductivity and dielectric properties of carbon-reinforced Si_3_N_4_ composite ceramics. 

Shukrullah et al. [159] used Fe_2_O_3_/Al_2_O_3_ as the catalytic support and discussed the ethylene flow rate and the entire process time for the growth of CNTs in a fluidized bed chemical vapor deposition (FBCVD) reactor at a temperature of 800 °C. The experimental results found that the best growth effect can be obtained when the ethylene flow rate is 100 sccm and the CVD process time is 60 min. At present, there are a large number of articles reporting CNTs grown by thermal CVD systems [160]. Many studies have shown that reaction acidity, chemical properties of catalyst nanoparticles, and carbon precursors are important parameters in the preparation of CNTs. 

Some researchers found that when the reaction temperature was below 400 ℃, the defect concentration of CNTs grown was very high, but the diameter was large, approximately 20 nm. However, when the reaction temperature was 400 ℃, the CNTs obtained had no obvious defects and were approximately 12 nm in diameter. If a single metal or bimetal catalyst is selected, WMCNTs can be synthesized at 600 ℃ in a CVD system with acetylene as precursor [161]. The catalysts were prepared by two methods, precipitation and solgel, with two different carriers, MgO and Al_2_O_3_. The highest yield of 800% was achieved by a two-component NiO/Co_2_O_3_/MgO catalyst. These MWCNTs were smooth and unidirectional. Their tube diameter was approximately 20 nm. The results show that the use of a single-component catalyst can promote the uniform growth of nanotubes and obtain smaller diameters; after being treated with concentrated nitric acid under reflux and washing conditions, the specific surface area of the nanotubes will increase and the average pore diameter will decrease. On the NiO/Co_2_O_3_/MgO catalyst, multi-walled carbon nanotubes with the largest specific surface area (305 m^2^/g) and average pore size (26 m^2^/g) with large structural adsorption characteristics were grown.

Li et al. [162] proposed that MgO is a good carrier for the preparation of carbon nanotubes by chemical vapor deposition. It can not only increase the yield, but also obtain carbon nanotubes with fewer impurities and controllable diameter by appropriately controlling the reaction temperature, catalyst, and gas flow rate and other reaction parameters. At the same time, MgO can be easily removed with weak acid without destroying the structure and quality of the product. If Mo salt is added as an auxiliary catalyst, the yield can be increased to approximately 120%.

There are some points that should be paid attention to when preparing carbon materials and graphene-like materials by the CVD method, such as the selection of precursors, deposition conditions, catalysts, and so on. The substrate has a certain relationship with the catalyst. In the process of preparing carbon materials and graphene-like materials by CVD, sometimes the substrate acts as the catalyst, and different choices will lead to differences in the structure and properties of the prepared materials. Therefore, summarizing and selecting the necessary preparation conditions is a major premise to determining the properties and application fields of materials. Table 2 lists the more basic conditions and the corresponding material selection. According to the different combinations of the three, the effects of different air velocity, temperature, pressure, and reaction time are different. Researchers should explore green and efficient preparation methods according to materials and their applications. Facing such a variety of choices, we need to summarize and explore more carefully.

## 4. Simulation of Synthesis Process of Carbon Materials Prepared by CVD

With the rapid growth of new synthetic materials, the development of low-dimensional nanomaterials with various morphologies has received significant attention. Among the many methods for preparing these nanomaterials, CVD is the most commonly used preparation method due to its simple operation, low cost, diversified product morphology, and high purity rates. However, the CVD method has many influencing factors, compromising control nanomaterial morphology. For nanomaterials, different morphologies have different properties and applications. Therefore, controlling the nanomaterial preparation process is extremely important.

**Figure 18:**
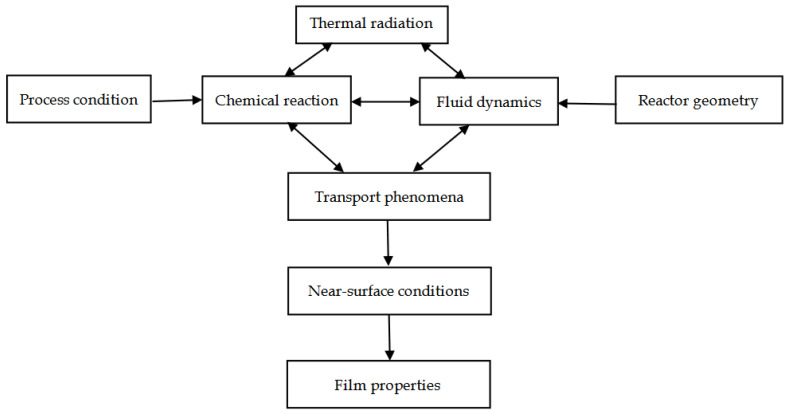
Simulation process [169].

The purpose of the CVD process and reactor simulations is to link the process conditions (gas flow, pressure, and temperature) and reactor structure to the properties of the film [169] (uniformity of film thickness, crystallinity, and chemical composition), as shown in Figure 18.

Simulations can illustrate the interactions between gas flow, heat and mass transfer, and chemical reactions in order to optimize CVD equipment and processes. Additionally, computational fluid dynamics (CFD) simulations can interpret experimental data and combine specific operating conditions with film properties. Furthermore, for complex physical and chemical changes in CVD reactors, valuable information can be obtained through simulation calculation analysis.

### 4.1. Simulation of Synthesis Process of Carbon Fibers Prepared by CVD

**Figure 19:**
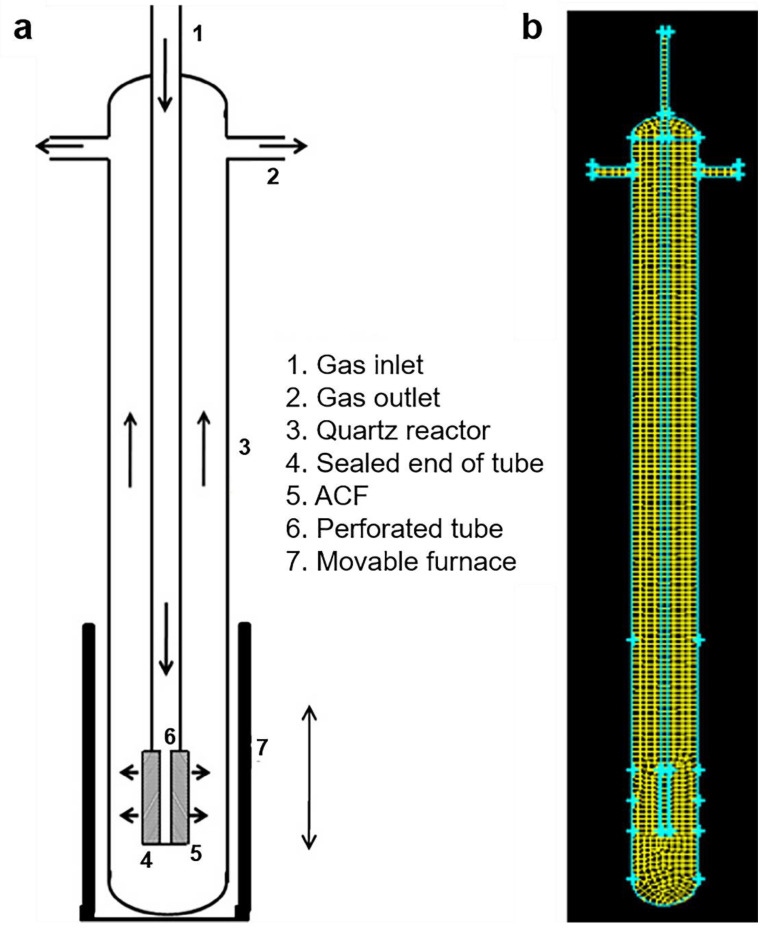
(**a**) Schematic of CVD reactor. (**b**) GAMBIT-mesh for simulation [170]. Copyright © 2021 Elsevier B.V. and the Institution of Chemical Engineers.

If the experiment is combined with computer simulation, it can not only verify the experimental conjecture, but also optimize the parameters of the existing experiments to achieve production optimization. Misshra and Verma used computational fluid dynamics to simulate the distribution of some reaction parameters of carbon nanofibers in a vertical chemical vapor deposition reactor, such as gas flow velocity, reaction temperature, and concentration release [170], as shown in Figure 19. Their simulation results show that computational fluid dynamics has the potential to optimize process operating conditions and that carbon nanofibers can be grown uniformly on a large scale in a vertical CVD reactor.

At present, the process simulation design of carbon fiber preparation by CVD is minimal. In order to expand the preparation methods and optimal parameters of preparing carbon fiber, computer simulation has become a necessary scientific research tool.

### 4.2. Simulation of Synthesis Process of Graphene Prepared by CVD 

Chemical vapor deposition is a potential method to produce high-quality and large-area graphene. However, as noted in the previous section, in the chemical vapor deposition system, the precursor, substrate, catalyst, and deposition conditions (temperature, pressure, time, air velocity, etc.) are all parameters affecting the deposition effect. Among them, hydrodynamics is one of the main factors, and computational fluid dynamics (CFD) is adopted. The growth temperature and speed of graphene can be simulated, and the optimal reaction conditions can be optimized or selected according to the simulation results.

**Figure 20:**
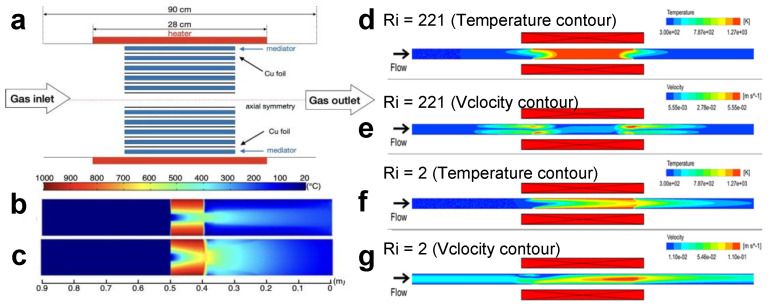
(**a**) Schematic diagram of CVD system simulation. Temperature distribution for the case of (**b**) nonporous and (**c**) 50% porous mediators [172]. Copyright © 2021 Elsevier B.V. and Taiwan Institute of Chemical Engineers. Contours of (**d**) temperature and (**e**) velocity distribution of Richardson number = 221 (Natural convection); (**f**) temperature and (**g**) velocity distribution of Richardson number = 2 (forced dominant-mixed convection). The furnace (red box) temperature is 1000 °C (1273 K) [173].

Hu et al. reported the relationship between gas flow rate and graphene structure quality and deposition uniformity [171]. The gas flow rate in the CVD furnace under different Cu substrate structures is simulated by CFD. Through simulation and experimental results, it is concluded that reducing the total gas flow rate can significantly improve the uniform distribution of graphene. Recently, He et al. [172] verified by computational fluid dynamics simulation that graphene films with high crystallinity and high conductivity can be synthesized if copper foil and carbon cloth are used as substrates (Figure 20a). The Brinkman equation is applied to describe the flow motion in the porous media. The commercial code COMSOL is used to couple the conservation equations. The quadratic shape function is used for velocity and the linear shape function for pressure and temperature.

The conservation of mass, momentum, and energy equations are expressed as:

Non-porous medial

(1) $$\nabla \cdot \left(\rho_{m}\vec{U}\right)=0$$

(2) $$\nabla \cdot \left(\rho_{m}\vec{U}\cdot \vec{U}\right)=\nabla \cdot \left[\mu_{m}\left(\nabla \vec{U}+{\left(\nabla \vec{U}\right)}^{T}\right)-\frac{2}{3}\mu_{m}\left(\nabla \cdot \vec{U}\right)I\right]-\nabla P+\rho_{m}\vec{g}$$

Porous medial

(3) $$\nabla \cdot \left(\rho_{m}\vec{U}\cdot \vec{U}\right)=\epsilon_{p}\nabla \cdot \left[\mu_{m}\left(\nabla \vec{U}+{\left(\nabla \vec{U}\right)}^{T}\right)-\frac{2}{3}\mu_{m}\left(\nabla \cdot \vec{U}\right)I\right]\;-\epsilon_{p}^{2}\nabla P-\epsilon_{p}^{2}\left({\mu \kappa }^{-1}\right)\vec{U}+\epsilon_{p}^{2}\rho_{m}\vec{g}$$

where $\rho_{m}$ is the density of gas mixture, $\vec{U}$ is the velocity of the mixed gas flow, $\mu_{m}$ is the viscosity of gas mixture, P is the pressure, $\epsilon_{p}$ is the porosity of mediator, κ the permeability of mediator, and $\vec{g}$ is the gravitational acceleration.

Through CFD simulation, we can see the temperature distribution under the conditions of porous and nonporous substrate (Figure 20b,c). The comprehensive study of optimization and multi physical field simulation shows that carbon cloth is an ideal spacer for graphene with high copper loading density. This enhancement is attributed to the uniform cross diffusion of reaction gas / species in the transverse and radial directions, resulting in uniform nucleation and equivalent kinetic growth. In the system, the capacity can reach 15.56 m^2^/h. Computational fluid dynamics simulation results show that porous media can improve the gas distribution between copper foils, balance the pressure between copper foils, and promote the high yield synthesis of graphene. It is concluded that under the conditions of a porous substrate, heat energy can pass through the copper foil and porous structure efficiently, which can improve production efficiency. This method provides an effective strategy for the synthesis of high-throughput and large-area deposited graphene films, which is conducive to the low-cost and multi-purpose applications of graphene based materials.

Fauzi et al. studied the influence of hydrodynamics through experimental and numerical methods [173]. In the experiment, two extreme cases of Richardson number with strong mixed convection (Ri = 2) and natural convection (Ri = 221) are selected (as shown in Figure 20d–g). It can be seen from Figure 20d,e a that when Ri = 221, the temperature in the heating zone in the furnace is evenly distributed, and the system is greatly affected by buoyancy effect. On the contrary, when Ri = 2, the temperature in the heating zone in the furnace is only 900 ℃ (Figure 20f), which is less affected by the buoyancy effect, and the flow rate is high (Figure 20g). It is concluded that at higher Reynolds number and lower Richardson number, the gas velocity is not affected by the buoyancy effect because the reflux is very small. At this time, the material residence time is short and the boundary layer is thinner, which can effectively avoid the formation of hydrocarbons and improve the quality of graphene.

In conclusion, porous media can be considered for the selection of substrate, which can improve the gas distribution between copper foils and promote the efficient synthesis of graphene. In the reaction system, attention should also be paid to the Reynolds coefficient. If the coefficient is small and the material residence time is short, high-quality graphene material can be formed. Through simulation, we can more intuitively understand which conditions have higher production efficiency, and even go straight to the depths of the problem to explore the main conditions affecting the reaction to optimize the parameters. For example, the uniform distribution of graphene can be significantly improved by reducing the total gas flow and adjusting the reflux coefficient and the effective heat distribution of porous substrate.

### 4.3. Simulation of Synthesis Process of Graphene-like Materials Prepared by CVD

Due to the excellent properties of graphene-like materials and their great application potential in a wide range of industries, it is a great challenge to explore a low-cost and scalable synthesis of high-quality graphene-like materials. The goal is to combine modern simulation technology with experiment to optimize reaction parameters and obtain the best reaction conditions, so as to prepare a batch of graphene-like materials with high quality and high yield. This will be an important research direction.

**Figure 21:**
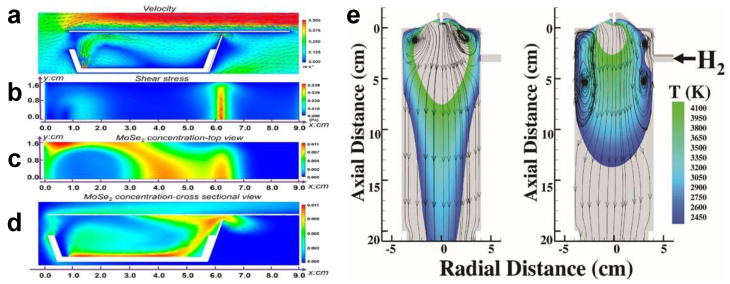
At t = 0.4 s, (**a**) velocity distribution and velocity vectors and (**b**) shear stress distribution over the growth substrate. (**c**) Top-view snapshot of MoSe_2_ concentration distribution. (**d**) Cross-section view of MoSe_2_ concentration. (**e**) The temperature distribution cut-off between 2300 and 4200 K as the BNNT growth temperature range (left, without hydrogen; right, with hydrogen).

Recently, Gao et al. successfully prepared GeSe_2_ nanofilms on mica substrate by employing chemical vapor deposition techniques [174]. Experimental results show that hydrogen is a key factor in the controlled growth of GeSe_2_ structure. They also revealed the relationship between sample thickness and distance through CFD simulations. It can be concluded that the closer to the cavity center, the higher the precursor concentration and thus the higher the nucleation density. In order to explore the influence conditions of MoSe_2_ growth by CVD, Zhou et al. summarized the growth simulation of standard APCVD and flip APCVD to obtain the simulation of evolutionary APCVD [175]. As shown in Figure 21a–d, the gas velocity distribution and shear force distribution, as well as the MoSe_2_ distribution on the cross section of the matrix and crucible are shown, respectively. By changing the relevant parameters, strengthening the mixing of precursors, reducing the nearby velocity shear rate, and adjusting the flow direction, inch scale monolayer MoSe_2_ can be successfully prepared.

Kim et al. analyzed the role of hydrogen in the growth of boron nitride nanotubes through thermodynamic equilibrium reaction calculation and numerical analysis of system heat flow [176]. It was found that the velocity amplitude of reverse flow near the wall in the presence of hydrogen is three times higher than that in the absence of hydrogen. When hydrogen is injected, a strong vortex will be generated in the system, making the precursor stay in the system for a long time, which can provide better conditions for the reaction system. The streamlines with arrows as well as the temperature distribution between nucleation and solidification of boron are shown in Figure 21e. Through this method, 12.6 g BNNTs can be obtained with high yield.

The preparation of graphene-like materials by CVD is a potential process. Due to the wide variety of graphene-like materials and different conditions, studies will get twice the result with half the effort if computer simulation technology is combined with experiment. The high-quality growth of graphene-like materials is explored through simulation technology, which opens up the market for the application of graphene-like materials.

### 4.4. Simulation of Synthesis Process of Carbon Nanotubes Prepared by CVD 

Simulation calculation analysis not only contributes to the improvement and optimization of the CVD reactor design but also explains the reaction mechanisms, including those of low-dimensional nanomaterials. Controllable equipment provides a theoretical basis [177], and CFD simulations can be divided into two main categories: chemical simulations of CVD, and the specific structural designs and its numerical simulations of the CVD reactor.

**Figure 22:**
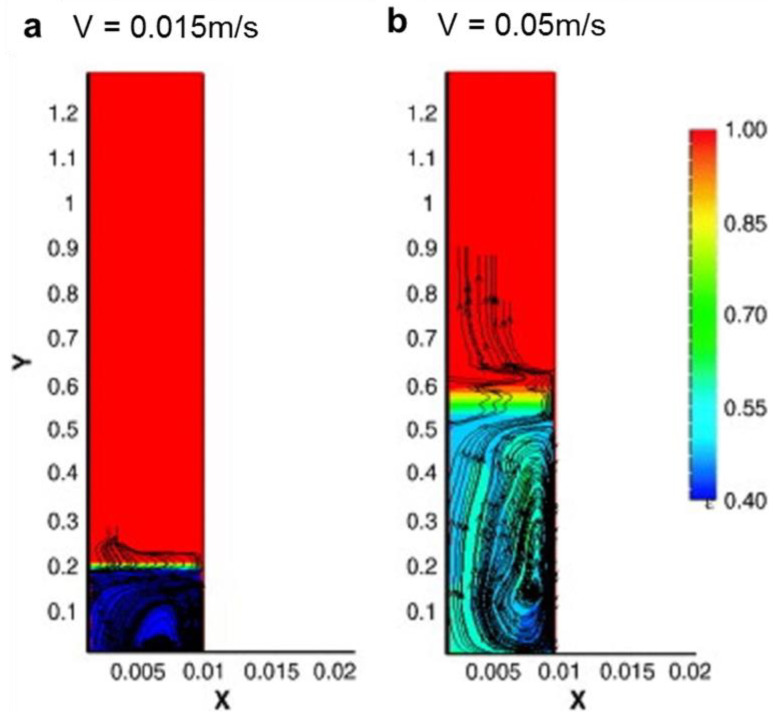
Void fraction distribution in the length of the reactor. The running speed and optimum speed are (**a**) V = 0.015 m/s and (**b**) V = 0.05 m/s [188].

The goal of the simulation was to illustrate the reaction mechanisms in the gas phase and surface reactions [177,178,179,180,181,182,183,184,185,186]. Endo et al. [187] proposed a CFD model that can predict the yield of carbon nanotubes through the catalytic decomposition of xylene in a CVD reactor. This model can predict the velocity and temperature distribution in the reaction zone and can analyze and obtain high-quality preparations. Moraveji et al. [188] studied MWCNT production using fluidized bed catalytic chemical vapor deposition, and a CFD method was used for simulating the hydrodynamics of the reactor and investigating the operational and best velocity for producing high-quality CNTs. These experiments revealed that a temperature of 900 °C, a CH_4_-to-hydrogen ratio of 1:4, and 0.02 m/s were the best parameters for MWCNT production using a fluidized bed reactor. The running speed and optimum speed obtained by MFIX simulation of computational fluid dynamics (CFD) program are 0.015~0.05 m/s. They also analyzed the porosity distribution in the reactor, as shown in Figure 22. When the flow rate is low, most of the reactants gather in the lower part of the reactor, from where it is difficult to leave the reactor. There are more and more catalytic particles, and the growth quality of carbon nanotubes is better. The simulation results are then applied to experiments with different temperatures and different gas compositions. The experimental results show that 900 ℃, 1:4 methane hydrogen ratio, and 0.02 m/s are the best parameters for the production of multi wall nanotubes in a fluidized bed reactor.

Seo et al. [189] used CFD to improve the uniformity of silicon carbide chemical vapor deposition and demonstrated that commercial CFD software can handle the entire reaction. Specifically, FactSage 6.2 and COMSOL 4.4 were used to predict the thermodynamic phases and deposition phenomena for SiC deposition, respectively. According to the simulation results, the process parameters were determined to optimize the growth conditions of a more uniform and effective SiC coating in the CH_3_SiCl_3_ (MTS) and hydrogen systems. CFD was then used to predict the growth rate of the film, and the prediction results were applied to the optimization of the film growth parameters to obtain a more uniform film thickness and a higher film growth rate. The correlation between the CFD model and the experimental results demonstrates the potential of CFD simulation in improving deposition conditions and optimizing hardware for better yields. A newly developed pulse plasma CVD method was used to successfully grow SWNTs with narrow chirality distributions at a relatively high yield, which was facilitated by improved control of SWNT incubation dynamics [190]. 

The simulation done by Kleijn and Hoogendoorn [191] and Kim et al. [192] mainly considered the fluid flow and transmission phenomenon inside the reactor under different process and operating conditions and reactor structures, while ignoring the chemical reactions in the CVD process.

**Figure 23:**
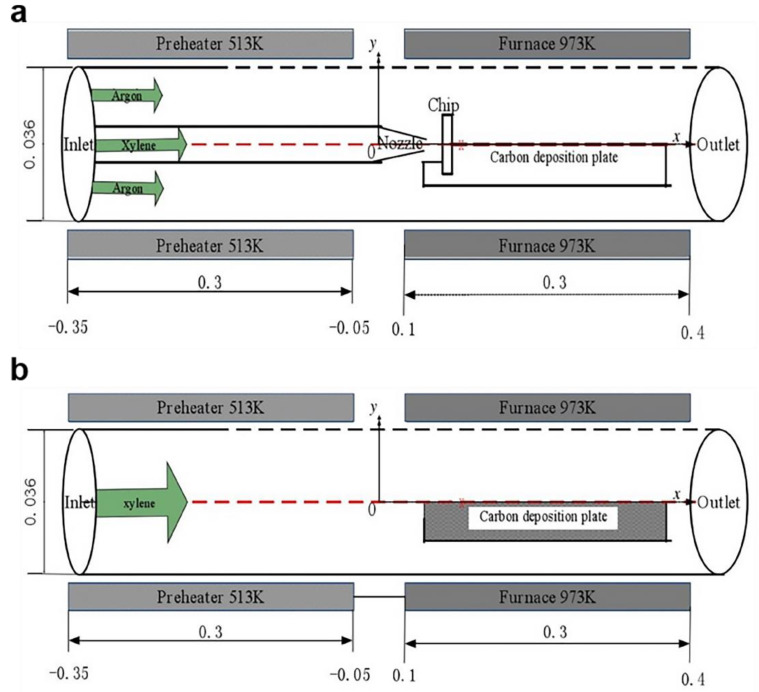
Schematics of chemical vapor deposition (CVD) reactor with nozzle (**a**) and CVD reactor without a nozzle (**b**) [193].

Min and Jiang [193] used CFD to simulate CNT synthesis by using a nozzle-type CVD reactor model. The numerical simulation reactor shown in Figure 23 consists of a quartz tube, a furnace, and a quartz platform. The quartz platform is placed in the quartz tube to provide a substrate for the growth of carbon nanotubes. The quartz tube is divided into two parts—the preheating furnace and the reaction furnace. Xylene evaporates in the first furnace and then enters into the second furnace. The corresponding chemical reaction is carried out in the second part, and the deposited carbon forms carbon nanotubes on the quartz platform. The model utilized the conservation equations for mass, momentum, and heat, and the mass fractions of the reactant, product species, and chemical reactions. Based on this model, the study simulated product distribution and the rate of the carbon deposition velocity distribution in a nozzle-type CVD reactor and CVD reactor without a nozzle and compared the carbon deposition variation rate of the nozzle-type CVD reaction at different temperatures. The simulation results showed that the nozzle-type CVD reactor was more efficient than the CVD reactor without a nozzle. Moreover, at 873, 923, 973, 993, and 1023 K, the carbon deposition rate increased as temperature increased.

**Figure 24:**
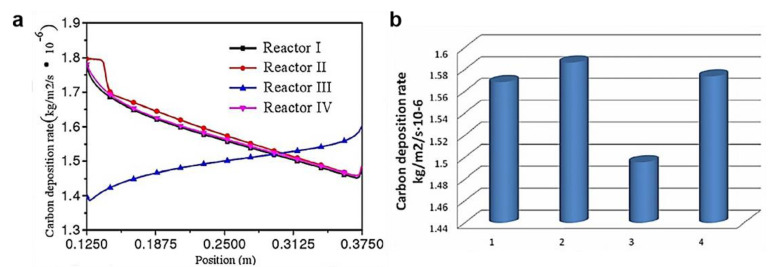
(**a**) Carbon deposition rates in four different structural reactors; (**b**) average carbon deposition rates in four different reactors [193].

The carbon deposition rate distribution curves of the four reactors with different structures were compared under the same external conditions, as well as the deposition rate curves of the four structures. The four structures are: (Ⅰ) without nozzle and chip structure; (Ⅱ) nozzle and chip structure; (Ⅲ) with nozzle and without chip structure; and (Ⅳ) with baffle and without chip structure. The results confirmed that the composition of the internal structure of the nozzle-type CVD reactor was beneficial in promoting increased carbon deposition (Figure 24).

Zhao et al. [194] also performed modeling to predict the effects of process parameters on carbon nanotube growth by CVD at ambient pressure using a simplified 2D geometry model. Acetylene was the precursor and argon was the diluent. The whole process was carried out at atmospheric pressure, and the precursor of the catalyst was the pure cobalt nitrate crystal (CO(NO_3_)_2_· 6H_2_O). The simulation used finite element methods and described the temperature, concentration, and velocity fields. The temperature field in the furnace region was simulated, and the positioning of the temperature field surface plot was presented and discussed. The results indicated that there was a steep thermal gradient in the furnace along the axial direction during the CVD process. However, there were equal temperature and concentration fields in the high-temperature region that were suitable for the growth of CNTs. When the volume ratio of acetylene to argon is 1:5, a large amount of carbon black and pyrolytic carbon are generated in the sediment. When the volume ratio of acetylene to argon is 1:10, carbon nanotubes with high purity can be obtained. Thus, the model was accurate enough to provide a guide for process optimization and assisted in detailing the principles of CVD processes for CNTs. The computer model was suitable for analyzing and studying the CVD process for the manufacturing of CNTs.

Xu’s team also used the CFD pre-processing software Gambit to build two two-dimensional models of gas flow field in the vertical carbon nanotube fiber reactor, which consisted of a straight inlet pipe model and a horn-shaped inlet pipe model, with a network that was divided to meet the simulation requirements [195]. Thus, a mathematical model of the gas flow field was established. Second, the flow field was numerically simulated using FLUENT calculation software, and the gas velocity field and temperature field distribution in the two reactors were obtained. Subsequently, the differential influence of the two different inlet pipe structures on the flow field was analyzed. Finally, the effects of the geometric parameters of the reactor on the flow field were assessed. A cylindrical inner sleeve was added inside the reactor, and gas property changes with temperature were incorporated into the model. The three geometries of the reactor were then studied using the controlled variable method. These analyses revealed a reflux zone near the outlet of the inner sleeve, which was consistent with the observed rising smoke, possibly explaining the phenomenon in which nanotubes tend to accumulate in the center of a reactor.

Recently, some CFD commercial software programs with CVD modules have improved, such as FLUENT, CFD-ACE, and CFX. These programs can simulate typical CVD phenomena, such as multi-component gas phase diffusion, heat conduction, and complex multi-component reactions, as well as gas phases and surface chemistry. Development of commercial software has further promoted simulation of the CFD process and equipment. CFD simulation is thus an auxiliary means for reactor design and process optimization, as well as a tool for rational interpretation of experimental data. Therefore, CFD simulation can be successfully used for design and optimization, providing a favorable method for computer-aided advanced CVD deposition [196,197,198].

It is found that high quality multi walled carbon nanotubes can be prepared when the hydrocarbon ratio is 1:4. When the precursor is acetylene, carbon nanotubes with high purity can be obtained when the volume ratio of acetylene to argon is 1:10. The nozzle furnace is conducive to more carbon deposition.

Table 3 lists the simulation results of some preparation processes. After the optimized parameters are obtained through the simulation, it can not only improve the quality of the product, but also improve the yield and growth size of the product, so as to provide theoretical support for the preparation of high-performance and large-scale products.

## 5. Summary and Future Directions

This paper summarizes the structure, properties, preparation, and modern computing technology of carbon materials—the application of computational fluid dynamics simulation in the preparation of materials. There are many kinds of carbon nano-materials and their structures have their own characteristics, and different structures will lead to special properties and affect the application field of the material. A major factor affecting the material structure is its preparation link. Different preparation methods and different conditions will have a great impact on the structure and properties of the material. This paper mainly introduces the preparation of carbon materials and graphene-like materials by CVD and the simulation of CVD system. The following is a summary:

1. The structural defects or doping of materials will have a certain impact on the properties of materials. The structure can be adjusted through the selection of preparation methods and the adjustment of process conditions to improve or inhibit some properties.
2. In addition to the selection of conventional precursors, substrate materials and catalysts, the reaction temperature, time, pressure, and atmosphere have an impact on the growth quality, yield, and growth size of the materials. Only by grasping the choice of precursor and other materials and controlling the process conditions can we obtain higher quality and high performance carbon materials.
3. Numerical simulation and analysis can provide valuable information for the complex physical and chemical changes in a chemical vapor deposition reactor. In the process of computational fluid dynamics simulation, the reaction parameters and reactor design can be optimized, and the temperature and velocity distribution of the reaction system can be simulated. Through the combination of simulation calculation and experiment, the improvement of production efficiency and production scale can be obtained.

With the rapid development of carbon nano-materials, demand for them is only increasing. Therefore, for the wide application of carbon nano-materials, the preparation process needs to keep pace to achieve high efficiency and high quality. The rapid development of computer technology undoubtedly contributes to the development of carbon nano-materials. Combining computer simulation with theory or experiment can optimize the existing preparation process; at the same time, it can verify reasonable guesses and realize the perfect combination of thinking and doing.

The development and study of carbon nano-materials with various morphologies has received significant attention, especially due to the urgent need for new synthetic materials. Presently, due to technical reasons, carbon nano-materials cannot be routinely used in industrial production due to difficulties in producing continuous high-quality carbon nano-materials in batches. Furthermore, the structure of the prepared carbon nanotubes cannot be arbitrarily controlled, as too many factors influence carbon nanotube quality and yield [201,202]. Among the various methods for preparing CNTs, the CVD method has attracted attention due to its simple operation, low cost, diversified morphology, and resulting high purity of the prepared CNTs. However, there are many experimental conditions for CVD, making it difficult to control the morphology of the CNTs. Thus, researchers have adjusted the morphology of CNTs by changing various experimental conditions to correlate experimental conditions with morphology, in order to better produce carbon nanotubes with the desired morphologies and properties. However, experiments cannot solely explain the effects of the local reactions on the reaction process. Combining computational analysis with experimental research provides a better method for determining the influence of various experimental conditions on the morphology of carbon nano-materials during the growth process. This allows the optimization of various process parameters, improving the reactor and preparing controllable high-quality carbon nano-materials. As a result, more in-depth research on carbon nano-materials will be important in the future—the reaction process and parameters are optimized by computer simulation technology, and the growth and structural changes of materials are regulated in combination with experiments, so as to achieve the purpose of application in different fields. 

## Figures and Tables

**Table 1:** Structure and properties.

Carbon Materials		Structural Features	Performance				Ref.
			Mechanical Properties	Electrical Properties	Magnetic	Optical Properties	
Carbon Fibers		Defective	×	-	-	-	[77]
		Modification	√	-	-	-	[78]
Graphene		Defective	×	-	√	-	[79,80]
		Doping	-	√	-	√	[81,82,83]
Graphene-like Materials	MoS_2_	Defective	×	√	-	-	[84,85,86,87]
		Doping	√	√		√	[88,89,90]
	WS_2_	Defective	-	×	√	-	[91,92,93]
		Doping	-	√	√	√	[94,95,96]
	h-BN	Defective	-	×	-	-	[97]
		Doping	-	-	√	-	[98,99]
Carbon Nanotubes		Defective	×	×	-	-	[100,101,102]
		Doping	√	√	√	√	[103,104,105,106]

Note: “√”: a positive change in this aspect; “×”: a negative change in this respect; “-”: no mention.

**Table 2:** Main conditions for preparation of carbon materials and graphene-like materials by CVD.

	Condition	Precursor	Substrate	Catalysts	Ref.
Material					
Carbon Fibers		CH_4_, C_2_H_2_, C_3_H_8_	Ni, Ni/Al_2_O_3_, SiO_2_/Si	SiC, Cu	[73,108,160,161,162]
Graphene		CH_4_/H_2_, CH_4_/H_2_/Ar,	Fe, Si/SiO_2_, Ni, Cu/W, Fe_2_O_3_/Si, Co/Cu	Cu, Ni, Co,	[122,163,164,165,166]
Graphene-like Materials	h-BN	(ClBNH)_3_, CH_4_, C_2_H_2_, (B_3_N_3_H_6_) (B_3_N_3_Cl_6_)	Pt, Si_3_N_4_/Si, Ni,	-	[124,125,126]
	WS_2_	WO_3_/S,	Al_2_O_3_	-	[126,167]
	MoS_2_	MoS_2_/S	Si/SiO_2_	-	[128]
Carbon Nanotubes		CH_4_, CO/CH_4_, Polypropylene, Acetonitrile, Aromatic molecules,	Cu	Ni, Fe_2_O_3_/Al_2_O_3_, Fe, Fe–Cu, Pt–W,	[130,131,132,133,134,154,155,156,157,158,168]

**Table 3:** Optimization of carbon nanomaterials properties and synthesis parameters by using computer simulations.

	Results	Production Quantity	Growth Velocity	Size	Ref.
Materials					
Carbon fiber		-	5 × 10^−13^ kg/m^2^·s	a large scale	[170]
Graphene		-	9.33 m^2^/h–17.88 m^2^/h	6–8 inch	[172]
		-	1.04 × 10^−7^ kg m^−2^·s^−1^	-	[199]
		-	1.5 µm·s^−1^	-	[200]
Graphene-like Materials	GeSe_2_	-	-	large-scale	[104]
	MoSe_2_	-	-	inch-scale	[175]
	h-BN	12.6 g	-	~7 nm	[176]
Carbon nanotube		350 mg h^−1^	-	-	[187]
		-	0.015~0.05 m/s	~188 μm	[188]

## Data Availability

Data sharing not applicable. No new data were created or analyzed in this study. Data sharing is not applicable to this article.
